# Molecular polymorphism, differentiation and introgression in the *period *gene between *Lutzomyia intermedia *and *Lutzomyia whitmani*

**DOI:** 10.1186/1471-2148-6-85

**Published:** 2006-10-27

**Authors:** Camila J Mazzoni, Nataly A Souza, Claudia Andrade-Coelho, Charalambos P Kyriacou, Alexandre A Peixoto

**Affiliations:** 1 Departamento de Bioquímica e Biologia Molecular, Instituto Oswaldo Cruz – Fiocruz, Rio de Janeiro, Brazil; 2 Departamento de Entomologia, Instituto Oswaldo Cruz – Fiocruz, Rio de Janeiro, Brazil; 3 Department of Genetics, University of Leicester, Leicester, UK

## Abstract

**Background:**

*Lutzomyia intermedia *and *Lutzomyia whitmani *(Diptera: Psychodidae) are important and very closely related vector species of cutaneous leishmaniasis in Brazil, which are distinguishable by a few morphological differences. There is evidence of mitochondrial introgression between the two species but it is not clear whether gene flow also occurs in nuclear genes.

**Results:**

We analyzed the molecular variation within the clock gene *period *(*per*) of these two species in five different localities in Eastern Brazil. AMOVA and Fst estimates showed no evidence for geographical differentiation within species. On the other hand, the values were highly significant for both analyses between species. The two species show no fixed differences and a higher number of shared polymorphisms compared to exclusive mutations. In addition, some haplotypes that are "typical" of one species were found in some individuals of the other species suggesting either the persistence of old polymorphisms or the occurrence of introgression. Two tests of gene flow, one based on linkage disequilibrium and a MCMC analysis based on coalescence, suggest that the two species might be exchanging alleles at the *per *locus.

**Conclusion:**

Introgression might be occurring between *L. intermedia *and *L. whitmani *in *period*, a gene controlling behavioral rhythms in *Drosophila*. This result raises the question of whether similar phenomena are occurring at other loci controlling important aspects of behavior and vectorial capacity.

## Background

The Phlebotominae sand flies *Lutzomyia intermedia *Lutz & Neiva 1912 and *Lutzomyia whitmani *Antunes & Coutinho 1912 are vectors of cutaneous leishmaniasis in Brazil. These are closely related species that can be only distinguished by a few morphological differences [1] and both show high anthropophily and reported natural infections with *Leishmania *in different regions of Brazil [2].

Despite their importance as vectors, only a handful of studies have been carried out in these two species using molecular techniques [3-6]. One of the most important findings from an epidemiological perspective is the evidence obtained for introgression between the two species using mitochondrial DNA [4]. This was particularly interesting because apparently, only lineages of *L. whitmani *sympatric with *L. intermedia *have been involved in cutaneous leishmaniasis transmission in the peridomestic environment [4], which suggests that genes controlling aspects of vectorial capacity could be passing from one species to the other. In fact, mitochondrial introgression has been reported in other sand fly species [7,8] suggesting that might be a common phenomenon in these insect vectors. However, because mitochondrial genes can introgress relatively easily between closely related species [9], it becomes important to examine whether introgression can occur with nuclear genes.

The *Drosophila period *(*per*) gene homologue was isolated in sand flies by Peixoto et al. [10]. This circadian clock gene was originally identified using mutagenesis by Konopka and Benzer [11], but is also known to control the differences in the "lovesong" rhythms between *D. melanogaster *and *D. simulans *[12], that are important to the sexual isolation between these two species [13-15]. In addition, *per *was implicated in the control of species-specific circadian mating rhythms in *Drosophila *and *Bractocera*, which might also constitute a reproductive isolation mechanism [16-18]. Thus *per *may possibly represent an example of a *Drosophila *speciation gene [19], and in fact it has been used as a molecular marker in a number of speciation and evolutionary studies, not only in *Drosophila *(reviewed in [20]) but also in other insects (e.g. [21]) including sand flies [22-24].

Because *per *controls the circadian clock in different insects [25], it is almost certainly involved in the rhythms of activity and biting of sand flies [26], which are very important to leishmaniasis transmission. In addition, *per *might be involved in reproductive isolation in sand flies, via mating rhythms, or via their "lovesongs" [2,27]. *per *is thus a particularly interesting marker, among the few available, for an introgression analysis in *L. intermedia *and *L. whitmani*. Evidence for introgression in *per *might suggest that gene flow between these two vector species is occurring at other genes controlling important aspects of behavior and vectorial capacity. It might also suggest that *per *does not have a strong role in their reproductive isolation. In the current study, we analyzed the molecular variation within the *per *gene of *L. intermedia *and *L. whitmani *in five different localities in Eastern Brazil.

## Results

### Polymorphism and divergence between *L. intermedia *and *L. whitmani*

**Figure 1:**
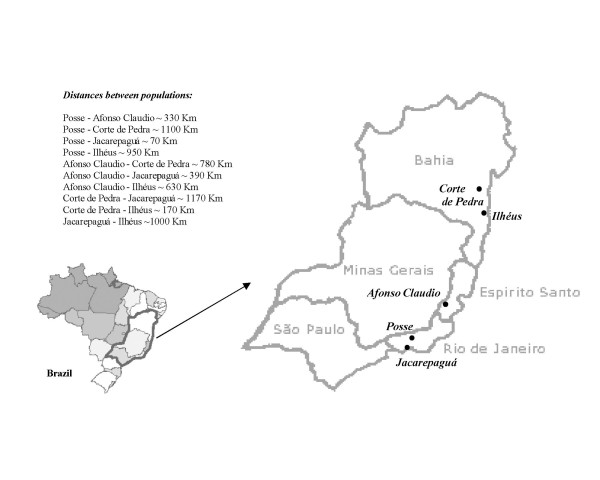
**Map of Southeastern Brazil**. The five localities (Afonso Claudio, Corte de Pedra, Ilhéus, Jacarepaguá and Posse) where the sand flies were collected are indicated on the map. Approximated distances between localities are also indicated.

**Figure 2:**
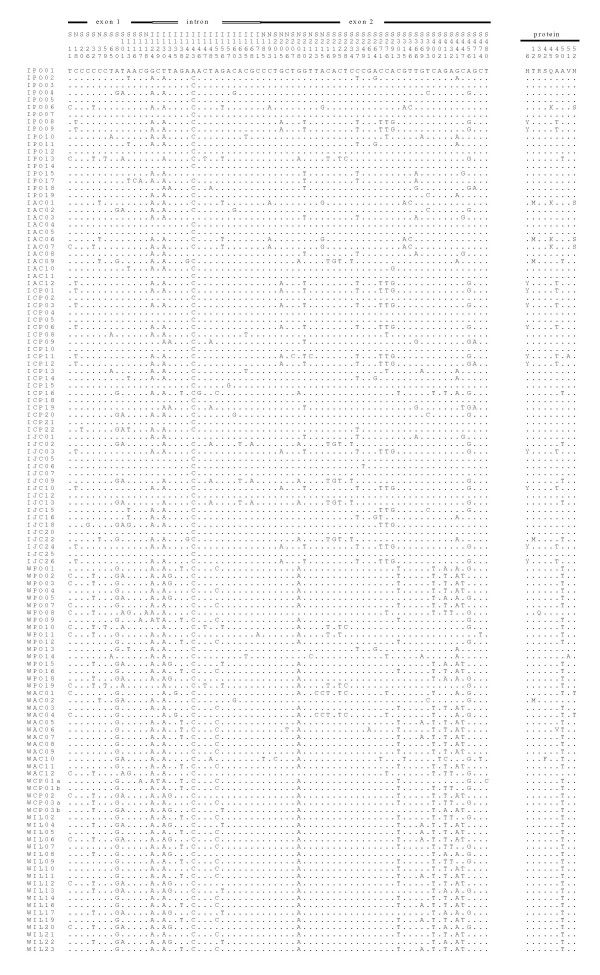
**Alignment of *L. intermedia *and *L. whitmani per *DNA and predicted amino acid sequences (variable sites only)**. IPO – *L. intermedia *from Posse; IAC – *L. intermedia *from Afonso Claudio; ICP *L. intermedia *from Corte de Pedra; IJC – *L. intermedia *from Jacarepaguá; WPO – *L. whitmani *from Posse; WAC – *L. whitmani *from Afonso Claudio; WCP – *L. whitmani *from Corte de Pedra; WIL – *L. whitmani *from Ilhéus.

A total of 68 sequences from *L. intermedia *and 53 from *L. whitmani *homologue to a fragment of the *period *gene were analyzed from populations of five localities in Eastern Brazil (Fig 1). The alignment of 72 variable sites is shown in Fig 2. Although most of the changes are either synonymous or occur within the 58 bp intron, non-synonymous substitutions are observed causing 9 amino acid differences among the sequences (Fig 2).

Table 1 shows the number of sequences of each population of the two species, the number of polymorphic sites (S) and the estimates of molecular polymorphism θ (based on the total number of mutations) and π. Table 1 also shows the Tajima's [28] and Fu & Li's [29] statistics. Within each species, all populations present similar levels of polymorphism with the exception of *L. whitmani *from Ilhéus, which seems to be less polymorphic than the others. This population was also the only one presenting a significant value in the Fu & Li test but only at the 5% level. Finally, the last column of Table 1 presents the recombination estimator γ [30] indicating that both species show evidence of intragenic recombination in the *per *gene.

**Table 1:** Molecular polymorphism in the *period *gene of *L. intermedia and L. whitmani*

population	*n*	*S*	θ	π	*D*_T_	*D*_FL_	γ
*L. intermedia*							

Posse	18	41	0.0251	0.0169	-1.3399^ns^	-1.1798^ns^	0.0594
Afonso Claudio	12	33	0.0225	0.0180	-0.9036^ns^	-0.6843^ns^	0.0187
Corte de Pedra	20	36	0.0209	0.0170	-0.7337^ns^	-0.8379^ns^	0.0099
Jacarepaguá	18	31	0.0191	0.0198	0.1347^ns^	-0.0250^ns^	0.0361
total	68	59	0.0266	0.0180	-1.0885^ns^	-0.9514^ns^	0.0353

*L. whitmani*							

Posse	17	35	0.0225	0.0215	-0.1811^ns^	-0.3380^ns^	0.0479
Afonso Claudio	12	36	0.0252	0.0215	-0.6606^ns^	-0.4393^ns^	0.0038
Corte de Pedra	5	17	0.0178	0.0183	0.2216^ns^	0.3069^ns^	0.0128
Ilhéus	19	14	0.0082	0.0123	1.8035^ns^	1.4992*	0.0195
total	53	50	0.0240	0.0183	-0.8154^ns^	-1.5360^ns^	0.0356

* p < 0.05; ns non-significant.

*n*, number of DNA sequences of each sample; S, number of polymorphic sites; θ, estimate of nucleotide diversity based on the total number of mutations; π, average heterozygosity based on the frequency of pairwise differences; *D*_T_, Tajima's *D *[28]; *D*_FL_, Fu & Li's *D *[29]; γ, estimator of recombination per base pair [30].

To investigate the level of intra and interspecific differences, initially an AMOVA was carried out as shown in Table 2. The results show a non-significant within species and a significant between species molecular variation at the *per *locus. Table 3 shows a more detailed analysis of the intraspecific differentiation among populations of *L. intermedia *and *L. whitmani*. None of the pairwise and overall fixation indexes (Fst) are significant in the case of *L. intermedia *and only one (Posse × Ilhéus) has a borderline significant value in *L. whitmani*. The results therefore show that no significant geographical heterogeneity was detected among the populations of the two species. The estimated number of migrants per generation, based on the overall Fst values, is 20.683 for *L. intermedia *and 23.125 for *L. whitmani*.

**Table 2:** AMOVA.

Source of Variation	d.f.	Sum of squares	Variance components	Percentage of variation
Among species	1	138.104	2.22072 Va *	33.37
Among populations within species	6	33.979	0.08996 Vb^ns^	1.35
Within populations	113	490.801	4.34338 Vc **	65.27
Total	120	662.884	665.406 Vt	

Fixation indices				

FSC (Vb/(Vb + Vc))	0.02029^ns^			
FST ((Va + Vb)/Vt)	0.34726 **			
FCT (Va/Vt)	0.33374 *			

* P < 0.05; ** P < 0.001; ns non-significant

**Table 3:** Pairwise and overall estimates of population differentiation between populations of *L. intermedia *and *L. whitmani*

	F_st_	Nm	P(F_st_)
*L. intermedia*			

IPO × IAC	0	∞	0.654
IPO × ICP	0	∞	0.651
IPO × IJC	0.0171	17.357	0.229
IAC × ICP	0.0407	5.886	0.117
IAC × IJC	0.0397	6.045	0.166
ICP × IJC	0.0002	1083.819	0.392
all populations	0.0119	20.683	0.231

*L. whitmani*			

WPO × WAC	0.024	10.271	0.188
WPO × WCP	0	∞	0.656
WPO × WIL	0.07	3.335	0.048
WAC × WCP	0.006	45.354	0.466
WAC × WIL	0.04	6.017	0.149
WCP × WIL	0	∞	0.643
all populations	0.0107	23.125	0.322

*F*_*st *_is the fixation index between populations inside each species [53]. *Nm *is the number of migrants per generation based on F_st_. The significance test of *F*_*st*_, *P*(*F*_*st*_), is based on 1000 permutations. IPO – *L. intermedia *from Posse; IAC – *L. intermedia *from Afonso Claudio; ICP *L. intermedia *from Corte de Pedra; IJC – *L. intermedia *from Jacarepaguá; WPO – *L. whitmani *from Posse; WAC – *L. whitmani *from Afonso Claudio; WCP – *L. whitmani *from Corte de Pedra; WIL – *L. whitmani *from Ilhéus.

Table 4 shows measures for DNA divergence between species (Dxy and Da), as well as the Fst and Nm values considering each species as a unique population. Dxy is the average number of nucleotide substitutions per site between alleles from two different populations and Da is the number of net nucleotide substitutions between two populations. Table 4 also shows the number of polymorphisms exclusive for each species (S_int _and S_whit_), the number of shared polymorphisms (Ss) and the number of fixed differences (Sf) between species. As one can note, there is a high number of shared polymorphisms between species, and no fixed differences between them suggesting either the persistence of ancestral polymorphisms or the occurrence of introgression. In fact, there is one shared haplotype between the two species (IPO13, WPO10 and WPO19) and three *L. whitmani *sequences (WAC02, WPO13 and WPO14) which show only one nucleotide difference to "typical" *L. intermedia *haplotypes (see also below).

**Table 4:** Divergence estimates between *L. intermedia *and *L. whitmani*.

D_xy_	0.0279 (0.0001)
D_a_	0.0095 (0.0013)
F_st_	0.3373 (P < 0.001)
Nm	0.4912
S_int_	18
S_whit_	27
S_S_	35
S_F_	0

*D*_*xy *_[54] is the average number of nucleotide substitutions per site between the two species and *D*_*a *_[54] is the number of net nucleotide substitutions per site. Both *D*_*xy *_and *D*_*a *_were calculated using Jukes & Cantor correction [55]. Standard deviations for *Da *and *Dxy *are between parentheses. *Fst *is the fixation index. The significance of *F*_*st*_, *P*(*F*_*st*_), is based on 1000 permutations as before and *Nm *is the estimated number of migrants per generation. *S*_*int *_is the number of sites that are polymorphic in *L. intermedia *and monomorphic in *L. whitmani*; *S*_*whit *_is the number of sites that are polymorphic in *L. whitmani *and *L. intermedia *in the first; *S*_*S *_is the number of polymorphic sites shared by the two species and *S*_*F *_is the number of fixed differences.

### Genealogy of *period *sequences

**Figure 3:**
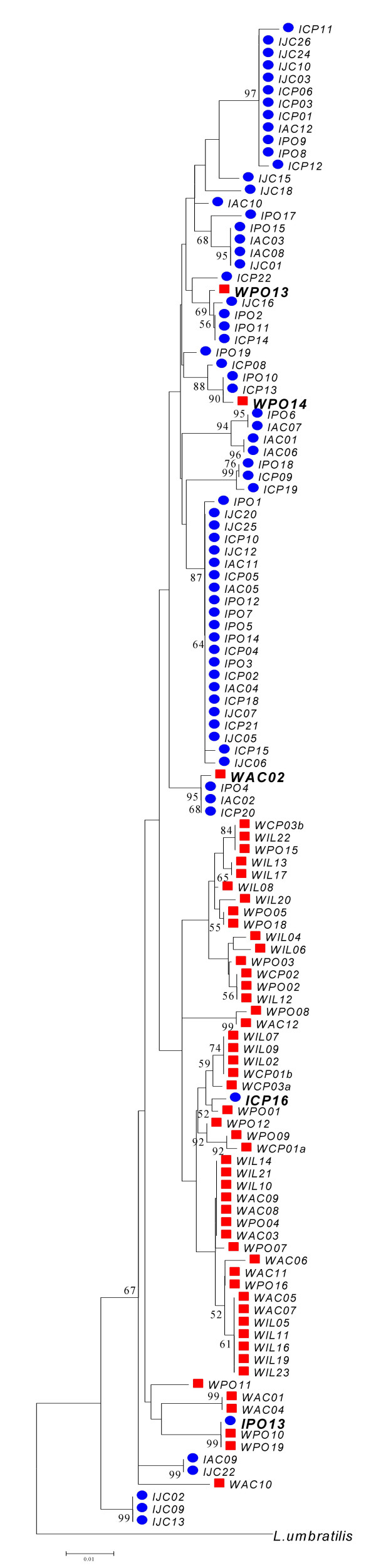
**Minimum Evolution tree**. A Minimum Evolution tree of the *period *gene sequences of Fig 2 using Close-Neighbor-Interchange Heuristic Search with an initial tree obtained by Neighbor-joining method, Kimura 2-parameter distance and 1000 bootstrap replications. *L. intermedia *sequences in blue circles and *L. whitmani *sequences in red squares. Putative introgressed sequences are highlighted with larger fonts.

A phylogenetic analysis of the *period *gene sequences from *L. intermedia *and *L. whitmani *was carried out with the Minimum Evolution method using the Kimura 2-parameter distance (Fig 3). A sequence from *L. umbratilis*, a related species from the same subgenus *Nyssomyia*, was used as outgroup [24]. The tree shows *L. intermedia *and *L. whitmani *as non-monophyletic. However, despite the low bootstrap values, which are below 50% in most cases, there is a large group that contains most *L. intermedia *sequences and a second large group with most *L. whitmani *sequences. A few other sequences are clustered outside these two main groups. It is interesting to note that there are three *L. whitmani *alleles (WAC2, WPO13 and WPO14) inside *L. intermedia *main group, as well as one *L. intermedia *allele (ICP16) inside the *L. whitmani *main group. In addition, a second *L. intermedia *allele (IPO13) is a shared haplotype between the two species as mentioned above. Again, the results suggest either the persistence of ancestral polymorphisms or the occurrence of introgression between the two species. Very similar results were obtained using the maximum likelihood algorithm as implemented in PAUP 4.0b10 software [31] (data not shown).

As mentioned before, there is evidence of intragenic recombination in the *per *gene fragment of both species (see Table 1) and for that reason the bifurcating tree shown in Fig 3 has to be viewed with caution, as different regions of the gene might have different phylogenetic histories [32]. Therefore, we constructed Minimum Evolution trees with the two most polymorphic non-recombining blocks of the *per *gene fragment identified using the Hudson and Kaplan [33] method available in the DNAsp 4.1 program [34]. We did not observed major changes in the genealogy of the *L. intermedia *and *L. whitmani per *sequences, especially regarding the five haplotypes (ICP16, IPO13, WAC2, WPO13 and WPO14) that clearly cluster with sequences of the other species (data not shown).

**Figure 4:**
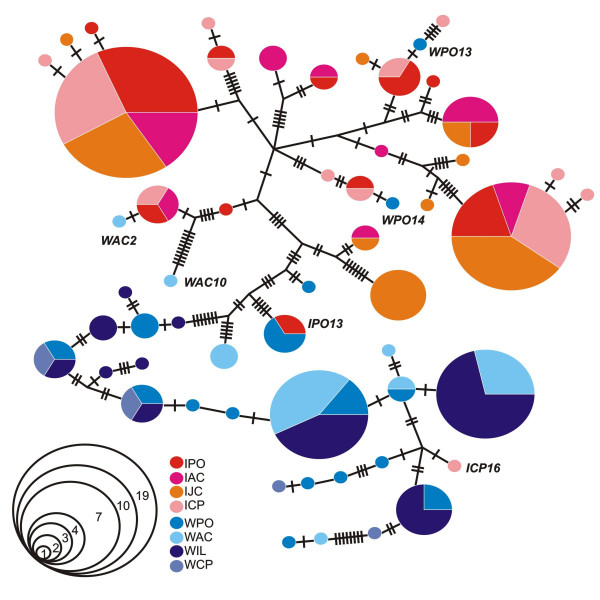
**Statistical parsimony network**. Each population is represented by a different color: PO – Posse, AC – Afonso Claudio, CP – Corte de Pedra, JC – Jacarepaguá, IL – Ilhéus. Each circle corresponds to a unique haplotype and is proportional to the number of sequences. The diagram on the left indicates the number of sequences depending on the circle size. Each cross bar represents one nucleotide substitution between two observed haplotypes. Putative introgressed sequences are indicated by their names.

Finally, a haplotype network was estimated from *per *sequences using statistical parsimony, as described by Templeton et al. [35] and implemented in the TCS1.21 software [36] (Fig 4). A small number of ambiguities were resolved as suggested by Crandall and Templeton [37]. The haplotype network shows connections between sequences from each species, separating most of the sequences of *L. intermedia *and *L. whitmani *in two groups. No intraspecific geographical structuring was found. Once again, some of the *L. whitmani *sequences (WAC2, WAC10, WPO13 and WPO14) appear more closely related to *L. intermedia *haplotypes. In addition, one *L. intermedia *allele (ICP16) is connected by a small number of mutations to some of the main *L. whitmani *haplotypes and IPO13 is a shared haplotype between the two species. These results confirm the same putative introgressed sequences indicated by the phylogenetic reconstructions.

### LD test of introgression

We tested the hypothesis of gene flow between *L. intermedia *and *L. whitmani *using a method based on linkage disequilibrium (LD) developed by Machado et al. [38]. In this test, *x *is the difference between the average LD found among all pairs of shared polymorphisms (DSS) between the two species and the average LD among all pairs of sites for which one member is a shared polymorphism and the other is an exclusive polymorphism (DSX). In case of gene flow *x *should tend to be positive [see [38] for more details].

Because of limitations on the total number of sequences that could be handled by the WH program we could not perform the simulations with all sequences. Therefore, we carried out the LD test of introgression between each pair of sympatric populations of *L. intermedia *and *L. whitmani *from the localities of Posse, Afonso Claudio and Corte de Pedra. The input files were prepared using the values of recombination and linkage disequilibrium calculated by the SITES program [30] for each population (data not shown). Although no significant values were found for the smaller samples of Afonso Claudio and Corte de Pedra, the results (Table 5) present evidence for introgression in the *period *gene in both directions (from *L. intermedia *to *L. whitmani *and vice-versa) in the locality of Posse.

**Table 5:** Linkage disequilibrium tests of gene flow between the two species.

	*L. intermedia*		*L. whitmani*	
Population	Obs.	Sim.	Obs.	Sim.
Posse	0.646	0.118	0.618	0.106
		(0.011*)		(0.018*)
Afonso Claudio	0.104	0.241	0.455	0.284
		(0.500)		(0.255)
Corte de Pedra	0.233	0.404	0.192	0.346
		(0.471)		(0.529)

In the first line for each species are the observed and mean simulated values of *x *(see text). The estimated probability of observing a simulated value higher than the observed value of *x *is presented in brackets below the mean simulated value of *x*; * less than 5% of simulated values higher than the observed value.

### Isolation with Migration model

To further examine the gene flow between *L. intermedia *and *L. whitmani *we used the IM software [39]. The Isolation with Migration model has six demographic parameters that include two migration rates, one for each population. The IM software estimates the posterior probability for each of the model parameters, fitting the Isolation with Migration model to the data. One of the assumptions of this model is that the loci studied do not have internal recombination. Therefore, we identified four different non-recombining blocks of our fragment of *per*, which were then treated as different loci in the analysis. The four-gametes test [33] implemented in DnaSP4.1 was used for the identification of possible recombination events. Since the program estimates parameters for a pair of closely related populations or species, all sequences of each species were used in the analysis as a single population. We performed MCMC runs using the IM software with different seed numbers, in order to guarantee convergence of the sample.

**Figure 5:**
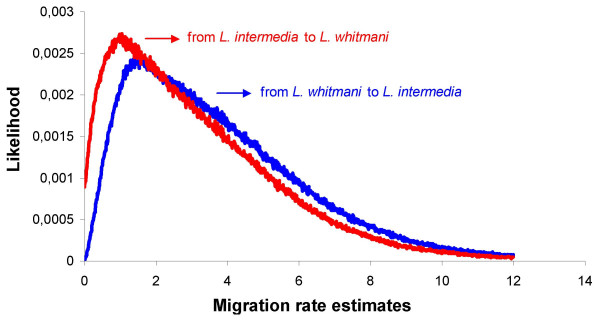
**Posterior distribution for migration estimates**. Posterior or likelihood distributions for migration rate estimates under the "Isolation with Migration" model [39]; *m*_1 _is the migration rate estimate from *L. whitmani *towards *L. intermedia *(in blue) and *m*_2 _from *L. intermedia *towards *L. whitmani *(in red).

Maximum likelihood estimates of migration parameters revealed a non-zero value for both species, *m*_1 _= 1.398 and *m*_2 _= 1.014 (*m*_1 _– from *L. whitmani *towards *L. intermedia*; *m*_2 _– from *L. whitmani *towards *L. intermedia*). Fig 5 shows the posterior distributions for migration rates and reveals a null probability for the absence of migration from *L. whitmani *towards *L. intermedia*. In addition, the absence of migration in the opposite direction is not included in the 95% confidence interval (values range from 0.222 to 8.898), thus supporting the presence of migration in both directions. The conversion of the migration rate estimate to population migration rate per generation (m_1 _and m_2_) is not accurate when the population size is based on a single locus. However, the average of the migrant number per generation for both species was very close to the Nm estimate based on Fst values (Nm ~0.49 in Table 4, m_1 _~0.52 and m_2 _~0.34).

## Discussion

There is some evidence that *L. intermedia *and *L. whitmani *might represent sibling-species complexes in Brazil. *Lutzomyia neivai *Pinto 1926, a sibling of *L. intermedia *is found in parts of Southern and Western Brazil and some other countries of South America [40]. The present study did not include populations of this species. In the case of *L. whitmani*, mitochondrial data [3,6] indicates three main lineages in Brazil: an Amazonian group, a North-South group and a Northeast group. We did not find strong evidence of a geographical differentiation in the *period *gene among populations of *L. whitmani *although one of the pairwise Fst comparisons (Posse × Ilhéus) was significant at the 5% level.

When we compare *L. intermedia *and *L. whitmani*, we find a highly significant Fst value (0.3373), which is however smaller than that observed for the *period *gene between sympatric siblings of *Lutzomyia longipalpis *(Fst = 0.3952) [23], a complex of cryptic species that are vectors of American visceral leishmaniasis. Therefore, despite the presence of diagnostic morphological characters to identify *L. intermedia *and *L. whitmani *[1] the level of molecular divergence in *period *is not as high as the cryptic *L. longipalpis *siblings.

Even though it is hard to distinguish introgression from the persistence of ancestral polymorphisms, a test of gene flow based on the signature introgression leaves on the patterns of linkage disequilibrium [38] as well as simulations that fit the "Isolation with Migration" model to the data suggest that *L. intermedia *and *L. whitmani *might be exchanging alleles at the *per *locus. This is further supported by the presence of shared haplotypes between the two species in Posse and very similar sequences in all sympatric populations. There is mounting evidence that introgression plays a major role in the evolution of closely related insect vector species. Introgression among vectors may have important epidemiological consequences. Gene flow in loci that affect vectorial capacity, such as those controlling host preference and susceptibility to parasite infection, can change the transmission patterns and consequently make the disease control a harder task. Introgression of genes that control adaptation to particular types of environment can also have a major impact on the spread of vector-borne diseases as was proposed for the major African malaria vector *Anopheles gambiae *[41]. The same can be said about genes controlling insecticide resistance. For example, Weill et al. [42] found a *kdr *mutation responsible for pyrethroid resistance in the Mopti form of *Anopheles gambiae*, a normally susceptible taxon of this species complex. Sequence analysis reveals that this resistant allele probably originates through introgression from the Savanna form.

Although *L. intermedia *and *L. whitmani *are closely related and only distinguished by a few morphological differences, they do show differentiation in some other important traits. For example, in Posse, one of the localities we studied, the two species show differences in abundance during the year. *L. intermedia *is more abundant in the summer while *L. whitmani *is more frequent in the winter months [2]. They also show differences in microhabitat preferences, *L. intermedia *being more common in the peridomestic area while *L. whitmani *is found mainly in the surrounding forest [2]. In addition, the two species show marked differences in their tendencies to bite humans in the early morning, with *L. whitmani *showing higher feeding rates than *L. intermedia *[26]. Therefore, despite the evidence of introgression in the *period *gene in this locality, there are important ecological and behavioral differences between the two species in Posse suggesting that gene flow is probably rather limited in loci controlling these traits. Hence, it is yet not clear whether introgression has played an important role in the evolution of *L. intermedia *and *L. whitmani*. Further work with other genes might help clarify the issue.

## Conclusion

Evidence for introgression between *L. intermedia *and *L. whitmani *obtained using mitochondrial DNA [4] seems to be corroborated by our data on the *period *gene, a nuclear marker. Nevertheless, considering that *period *is potentially involved in reproductive isolation and might be, therefore, less prone to introgression than the "average" gene [43], it is possible that much higher levels of gene flow between the two species occur at other genes. It might, on the other hand, suggest that this behavioral gene, or at least the fragment we analyzed, did not play a role in speciation between *L. intermedia *and *L. whitmani*. In fact the same has been suggested for some *Drosophila *species [44] despite *per*'s role controlling lovesong and mating rhythm differences between *D. melanogaster *and *D. simulans *[13-16].

Although the evidence for introgression in the *per *gene between *L. intermedia *and *L. whitmani *is not overwhelming, it does indicate the need to extend this analysis to other loci in the future. We are currently isolating new molecular markers in the two species to carry out a multi-locus approach [39] that might help determining how much variation in gene flow and differentiation there is across the genome of these two very important leishmaniasis vectors.

## Methods

### Sand fly samples

Sand fly samples used in this work were all the F1 generation from wild collected females from the Brazilian localities of Posse (Petrópolis, Rio de Janeiro State, 22°30'S 43°10'W), Jacarepaguá (Rio de Janeiro, Rio de Janeiro State, 22°55'S 43°21'W), Afonso Claudio (Espírito Santo State, 20°04'S 41°07'W), Corte de Pedra (Presidente Tancredo Neves, Bahia State, 13°27'S 39°25'W) and Ilhéus (Bahia State, 14°50'S 39°06'W). *L. intermedia *and *L. whitmani *were identified according to Young and Duncan [1]. The progeny of each wild caught female was raised separately according to Souza et al. [45] and only one F1 male of each female was used for the molecular analysis, which included 68 individuals of *L. intermedia *(12 from Afonso Claudio, 18 from Posse, 20 from Corte de Pedra and 18 from Jacarepaguá) and 51 individuals of *L. whitmani *(12 from Afonso Claudio, 17 from Posse, 3 from Corte de Pedra and 19 from Ilhéus). Note that, although the distribution of the two species shows considerable overlap in Eastern Brazil, in many localities only one species is found or is far more abundant than the other. There are also seasonal and microhabitat differences in abundance between them in areas of sympatry [2].

### DNA methods

Genomic DNA was prepared according to Jowett [46] with slight modifications and the PCR was carried out for 30 cycles at 95°C for 30 sec, 60°C for 30 sec and 72°C for 30 sec, using Abgene, Amersham Biosciences or Biotools reagents according to manufacturers directions. The *per *primer sequences are: 5llper2: 5'-AGCATCCTTTTGTAGCAAAC-3' (forward) and 3llper2: 5'-TCAGATGAACTCTTGCTGTC-3' (reverse). These primers amplify a 486 bp fragment of the sand fly *per *gene homologue that includes part of the PAS/CLD domain, an intron (58 bp) and the beginning of the *per*^*S *^domain [24]. The amplified fragments were cloned using the *pMOSBlue *blunt ended cloning kit (Amersham Biosciences) and plasmid DNA preparation was carried out using the "Flexiprep" Kit (Amersham Biosciences). Cloned PCR fragments were sequenced at Fundação Oswaldo Cruz and at University of Leicester using ABI 377 sequencers. With the exception of two *L. whitmani *individuals from Corte de Pedra (see below), only one sequence of each sand fly (representing one of the two possible alleles) was used in the analysis but an average of three sequences per individual were obtained in order to check possible PCR induced mutations. In addition, PCR fragments were also sequenced directly in some cases for the same reason. In the case of the two *L. whitmani *mentioned above 6 and 9 clones were sequenced respectively from specimens WCP01 and WCP03 to determine both alleles simply to increase the size of this small sample.

Negative controls were performed for all amplification reactions. In addition, PCR, cloning and sequencing were repeated for two individuals to confirm putative introgressed sequences and to exclude the possibility that they were the result of PCR contamination. Finally, for at least two individuals with putative introgressed sequences, we could define the other allele from additional clones (not included in the analysis), which showed to be typical of the species, indicating no identification problems.

The sequences were submitted to GenBank (accession numbers AY927062 to AY927182).

### Sequence analyses

The preliminary sequence editing was carried out using the Wisconsin Package Version 9.1, Genetics Computer Group (GCG), Madison, and ClustalX [47] was used to perform the multiple alignment. Analyses of population polymorphisms and differentiation between populations were carried out using DNAsp4.1 [34] and ProSeq [48] softwares, while Arlequin v. 2.0 [49] was used for an analysis of molecular variance (AMOVA) between populations. The Minimum Evolution phylogenetic tree was constructed using MEGA 3.1 software [50]. The haplotype network was estimated using TCS1.21 [36]. Recombination and linkage disequilibrium analyses were performed using the DNAsp4.1 and SITES program [30]. Linkage disequilibrium simulations were carried out by the WH program [51,52] and Markov Chain Monte Carlo (MCMC) simulations of the isolation with migration model were performed using the algorithm implemented in the IM program [39].

## Authors' contributions

CJM generate and analyzed all the data and drafted the manuscript. NAS and CAC collected and maintained sand fly samples. CPK helped to write the manuscript and supervised CJM during her stay in Leicester. AAP is the principal investigator, participated in its design and coordination, and helped to write the manuscript. All authors read and approved the final manuscript.
